# Cytological Study of *Cypripedium japonicum* Thunb. (Orchidaceae Juss.): An Endangered Species from Korea

**DOI:** 10.3390/plants10101978

**Published:** 2021-09-22

**Authors:** Bokyung Choi, Geun-Hye Gang, Hyeonjin Kim, Hyejoo Byun, Minyeong Kwak, Soonku So, Hyeon-Ho Myeong, Tae-Soo Jang

**Affiliations:** 1 Department of Biological Science, College of Bioscience and Biotechnology, Chungnam National University, Daejeon 34134, Korea; cbokyung@cnu.ac.kr (B.C.); hyeonjin.k9924@gmail.com (H.K.); imbkbyun@gmail.com (H.B.); kmy705@naver.com (M.K.); 2 Plant Conservation Center, Korea National Park Research Institute, 2 Baengnyeonsa-gil, Seolcheon-Myeon, Musu-gun 55557, Jeollabuk-do, Korea; mama1446@knps.or.kr; 3 Korea National Park Research Institute, 171 Dangu-ro, Wonju-si 26441, Gangwon-do, Korea; ssk822@knps.or.kr

**Keywords:** *Cypripedium japonicum*, endangered species, haploid chromosome number, karyotype, genome size

## Abstract

Changes in chromosome number and karyotype evolution are important to plant diversification, as they are both major drivers of speciation processes. Herein, chromosome number, karyotype, and genome size of the Korean lady’s slipper orchid *Cypripedium japonicum* Thunb., an endangered species, were investigated in natural populations. Furthermore, all cytological data from this species are reported herein for the first time. The chromosome number of all investigated *C. japonicum* plants was diploid (2*n* = 2*x* = 22), with *x* = 11 as base chromosome number, whereby the species can now be clearly distinguished from the Japanese lady’s slipper orchid. The karyotypes of all studied individuals were of similar length, symmetrical, and rather unimodal. Flow cytometry of the *C. japonicum* revealed that the genome size ranged from 28.38 to 30.14 pg/1C. Data on chromosome number and karyotypes were largely consistent with previous results indicating that Korean (*x* = 11) populations of *C. japonicum* are more closely related to Chinese populations (*x* = 11) compared to Japanese (*x* = 10) populations. These comprehensive cytological results will benefit the efforts to discriminate the geographically isolated and endangered Eastern Asian (China, Japan, and Korea) lady’s slipper orchid species.

## 1. Introduction

The genus *Cypripedium* L. (Orchidaceae Juss.) comprises approximately 50 species of terrestrial herbs primarily distributed in the temperate regions of the northern hemisphere, including Asia and North America [1]. *Cypripedium japonicum* Thunb., commonly known as lady’s slipper orchid, is a rare to extremely rare perennial herb found across East Asia (China, Japan, and Korea) [2,3,4,5,6,7]. The species is characterized by having a large and attractive flower and two subopposite fan-shaped leaves [1,5]. The species has been globally categorized as endangered based on the International Union for Conservation of Nature (IUCN) Red List of Threatened Species [2], and it is nationally listed as vulnerable in Japan [4] and critically endangered in Korea, respectively [3,5]. The species is increasingly becoming rare due to over-collection/over-exploitation from its natural habitats for horticultural and medicinal purposes [2,3,4,5], and protection and conservation of natural individuals/populations of *C. japonicum* is crucial both locally and globally [2,3,4]. Despite the increasing importance of the conservation status, biological studies of the *Cypripedium* species have been focused on population genetics [5,6,8], pollination biology [9] and reproductive characteristics [7], and no comprehensive cytological analyses have been performed to date for this orchid. The genus *Cypripedium* has two base chromosome numbers: *x* = 10 (2*n* = 20 and 30) and 11 (2*n* = 22), with the most frequently encountered chromosome number being 2*n* = 20 in *Cypripedium* species [10,11]. In *C. japonicum,* diploids were reported from Japanese (2*n* = 20; [10,12,13]) and Chinese populations (2*n* = 22; [14]) with different base chromosome numbers. To date, however, no chromosome number and karyotype data of *C. japonicum* from Korean populations are available.

Changes in chromosome number (aneuploidy/dysploidy or polyploidy) and structure (inversions, translocations, additions, deletions) play a crucial role in plant evolution and diversification [15,16,17]. Cytological research, including karyotype analysis and genome size estimation, is considered as an important and useful approach in evolutionary studies of angiosperms [18,19,20,21,22,23,24]. Previously published genome size values within genus *Cypripedium* showed a 10.83-fold difference, ranging from 4.14 pg/1C in *C. molle* to 44.84 pg/1C in *C. acaule* [24]. However, to date, nuclear DNA content data of *Cypripedium* species assessed using flow cytometry analysis are available only for nine species out of fifty species in total, and it is still not available for *C. japonicum* [24]. As chromosome number, karyotype structure, and genome size data have been proved highly informative taxonomically—particularly within the *Cypripedium* genus [10,12,13,14]—these data should be obtained from natural populations of Korean lady’s slipper orchids.

Thus, the goals of this study were: (1) to establish the chromosome number and the haploid chromosome-size variation as well as the karyotype structure of *C. japonicum* from multiple natural populations in Korea; and (2) to provide data on the C-value to reveal possible patterns of variation in genome size for this species.

## 2. Materials and Methods

### 2.1. Cytological Analysis

**Figure 1:**
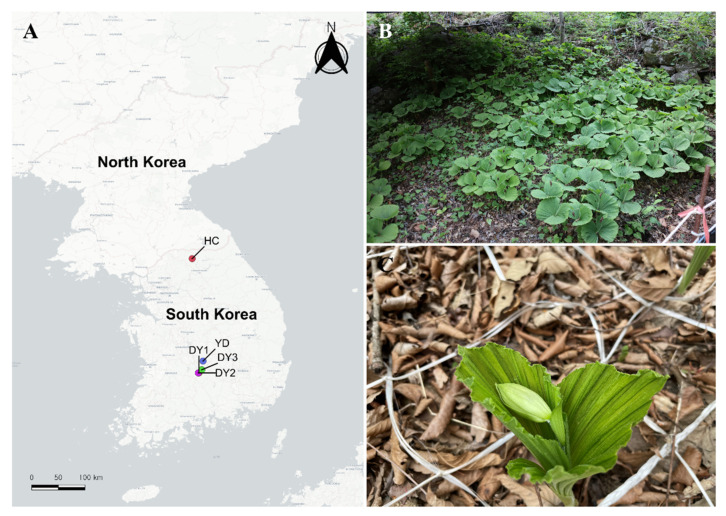
Sampled populations of *Cypripedium japonicum* in Korea (**A**), DY1, DY2, and DY3 (Mt. Deokyu population), YD (Youngdong-gun population), HC (Hwacheon-gun population), detailed information is given in Table 1. (**B**) Natural habitat of Hwacheon-gun population (HC), (**C**) Habit, unopened flower bud for meiotic analysis.

Young flower buds from 11 individuals were collected from 5 natural populations of *Cypripedium japonicum* Thunb. (Table 1; Figure 1) with special permission from the Korean Government (permit no. 2021-17 and 2021-18). Pollinia, including numerous pollen grains, is a specific characteristic of the Orchidaceae family [25], and thus chromosome numbers were determined from meiotic cell divisions in pollen mother cells of pollinia due to the lack of typical root meristems in *C. japonicum* (Figure 1C). The samples were immediately fixed in freshly prepared fixative (3:1 ethanol: acetic acid) and stored at −20 °C until subsequent use. Chromosome numbers were determined using the standard Feulgen staining technique according to Choi et al. [20]. In brief, young pollinia of flower buds were hydrolyzed in 5 N HCl at 25 °C for 30 min and immediately stained with Schiff’s reagent (Merck KGaA, Darmstadt, Germany) for 60 min. Squash preparations were made in a drop of 60% acetic acid. At least five well-spread chromosome plates were analyzed for each individual using a light microscope (Olympus BX-53, Tokyo, Japan) and photographed using a digital camera (Olympus DP-74, Tokyo, Japan), following the method by Choi et al. [21]. A minimum of three well-spread chromosome plates was selected for karyotype analysis and chromosome-size measurement [23].

### 2.2. Genome Size Measurement

The genome size of 20 individual *C. japonicum* plants was determined by flow cytometry with *Vicia faba* L. “Inovec” (13.45 pg/1C) as an internal reference standard [26,27]. Approximately 20–30 mg of fresh leaves from each sample were co-chopped with an internal reference standard using Otto’s buffer I [28], and isolated nuclei were stained using propidium iodide with RNase IIA (both at 50 µg/mL) solution for 5 min at 25 °C, and analyzed using a Sysmex CyFlow cytometer (Sysmex Partec GmbH, Görlitz, Germany). The methodology used for the measurement of the genome size was as described by Choi et al. [22]. The 1C value was calculated as indicated by Doležel et al. [27]: sample peak mean/standard peak mean × 1C DNA content of standard (pg). Genome size measurements were performed in triplicate for each *C. japonicum* individual.

## 3. Results and Discussion

### 3.1. Chromosome Numbers and Karyotype Structure of Cypripedium japonicum Thunb.

**Figure 2:**
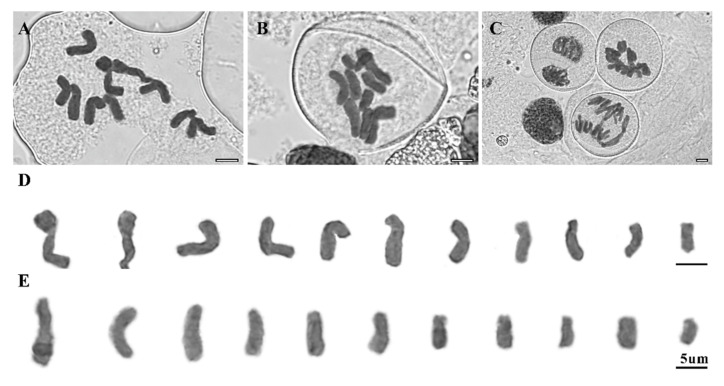
Meiotic chromosomes and karyotypes of *Cypripedium japonicum*. (**A**,**C**,**D**) collection number (population_individual): DY1_5, (**B**,**E**) collection number (population_individual): DY2_2, detailed information is given in Table 1.

The chromosome number and the karyotype of Korean populations of *C. japonicum*, the endangered Korean lady’s slipper orchid, are reported here for the first time (Table 1 and Table 2; Figure 1 and Figure 2). The 11 individuals (sampled from five populations) were all diploids with *n* = 11 (2*n* = 22; Figure 2A,B,D,E), as previously reported for Chinese populations [12], although *n* = 10 (2*n* = 20) was also found by several studies from Japan [10,12,13].

The representative karyotypes with detailed chromosome size are presented in Figure 2D–E and Table 3. No meiotic abnormalities, such as laggards or bridges during cell divisions, were found in any of the analyzed *C. japonicum* individuals (Figure 2C). 

The previously reported base chromosome number for *Cypripedium* was constant (*x* = 10), with diploids being the most common among the species followed by triploids (Table 2; [29,30,31,32,33]), except for *C*. *macranthos* that presents 2*n* = 22 [11]. The Chinese and Korean populations of *C. japonicum* clearly showed 2*n* = 22 (*n* = 11; Figure 2D–E; [14]), whereas those from the Japanese populations had 2*n* = 20 (Table 2; [10,12,13]). In *Cypripedium*, *C. japonicum* and *C. macranthos* had two base chromosome numbers, and this was not found in the rest of the *Cypripedium* species studied to date (Table 2). The difference in base chromosome number between Chinese–Korean (*x* = 11) and Japanese (*x* = 10) populations strongly indicated that the Korean populations of *C. japonicum* are more closely related to Chinese populations compared to the Japanese populations based on base chromosome numbers, and this result was consistent with population genetic analyses using ISSR and SCoT markers [5,6]. Thus, further studies, including physical mapping of chromosomes using FISH (fluorescence in situ hybridization) as well as molecular phylogenomics and population genomics of *C. japonicum* and its closely related species, could help clarify the evolutionary history of *C. japonicum* [19,34,35,36,37,38,39,40].

The karyotypes of the *C. japonicum* individuals investigated herein were all very similar, containing meta- and submetacentric chromosomes (Figure 2D–E). The total haploid length of chromosomes ranged from 87.04 µm (accession number: DY1_5) to 102.07 µm (accession number: DY1_1). Concomitantly, individual chromosome lengths ranged from 4.26 to 4.58 µm in the shortest and from 12.33 to 14.57 µm in the longest chromosomes (Table 3). The ratio of the longest to shortest chromosome (RI) ranged from 1.69 to 3.51, with a similar asymmetry index (59.9–66.7 %; Table 3).

Karyotype analysis relies on the identification of individual chromosomes and has been a challenge for most non-model plants, especially while conducting direct comparisons for chromosome size and karyotype formula among related species [17,22,34]. The newly counted chromosomes and karyotypes for Korean endangered lady’s slipper orchids presented here are against the deviating counts of 2*n* = 22 (14 m + 6 sm) from Japanese populations [13], implying the likely involvement of dysploidy or aneuploid numbers. However, karyotypes in the data reported herein were constant among populations, as previously observed in Chinese populations [14] and as suggested by the lack of genetic variation either within or among populations of *C. japonicum* in Korea [5,6].

### 3.2. Genome Size Estimation in Korean C. japonicum

**Figure 3:**
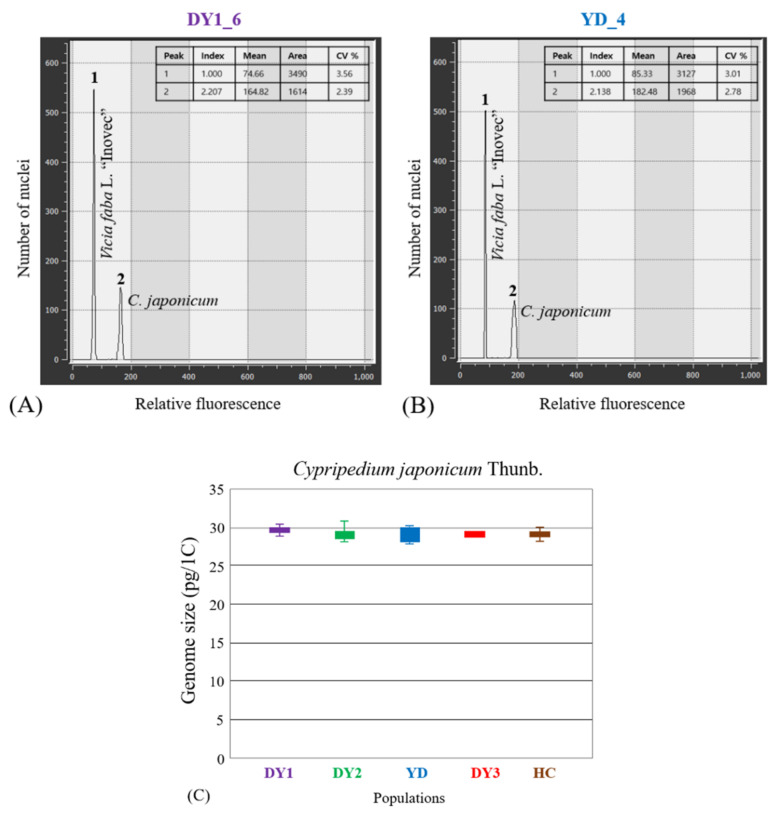
Fluorescence histogram results of *Cypripedium japonicum* with an internal reference standard (*Vicia faba* L. “Inovec”: 13.45 pg/1C). (**A**) DY1_6 (population_individual; Mt. Deokyu population), (**B**) YD_4 (population_individual; Youngdong-gun population), (**C**) Distribution of genome size data [1C (pg)] in the investigated five natural populations. Detailed population information is given in Table 1.

The 1C DNA contents of the investigated plants are shown in Table 1 and Figure 3, which are reported in this study for the first time. DNA content analysis revealed well-defined peaks for all samples (Figure 3A,B). As measured by flow cytometry using propidium iodide, the genome size of the analyzed *C. japonicum* individuals ranged from 28.38 pg/1C to 30.14 pg/1C (Table 1; Figure 3C). The C-values within the genus *Cypripedium* have been reported ranging from 4.14 pg/1C in *C. molle* to 44.84 pg/1C in *C. acaule*, in spite of which, they all had the same chromosome number, 2*n* = 2*x* = 20 (Table 2; [22]). The underlying reason for genome size differences among the *Cypripedium* species might be the differential accumulation of non-coding repetitive DNAs, as reported in other plant groups (Figure 3C; [19,40,41]). An understanding of chromosomal variation among populations concomitant with changes in genome size may provide novel insights into the ecological conservation status for the extremely endangered/threatened taxa, as reported for other genera of Orchidaceae [30,42,43].

## 4. Conclusions

This study presents the first comprehensive cytological analysis of meiotic chromosome number, karyotype, and nuclear DNA content of the endangered orchid *C. japonicum* from multiple Korean populations. In light of our results, the data on chromosome numbers and karyotypes are largely consistent with the previous population genetic study indicating that Korean populations (*x* = 11) of *C. japonicum* are more closely related to Chinese populations (*x* = 11) than they are to Japanese (*x* = 10) populations. The cytogenetic study can be beneficial to the discrimination of geographically isolated Eastern Asian (China, Japan, and Korea) endangered lady’s slipper orchids, and provide biological background for conservation. To protect and enhance the conservation status of this endangered species, further molecular cytogenetic and phylogenomic studies, including DNA sequencing based on additional population genetic markers, are needed using a larger number of individuals from multiple populations of East Asian orchids.

## Figures and Tables

**Table 1:** Plant material used for cytogenetic analyses in *Cypripedium japonicum*.

Collection Number (Population_Individual)	Locality; Collector	Chromosome Number	Genome Size 1C ± S.D. (pg)
DY1_1	Mt. Deokyu, Muju-gun, Jeollabuk-do; MHH	2*n* = 22	29.58 ± 0.59
DY1_2	Mt. Deokyu, Muju-gun, Jeollabuk-do; MHH	-	29.27 ± 0.49
DY1_3	Mt. Deokyu, Muju-gun, Jeollabuk-do; MHH	-	29.59 ± 0.35
DY1_4	Mt. Deokyu, Muju-gun, Jeollabuk-do; MHH	-	29.86 ± 0.23
DY1_5	Mt. Deokyu, Muju-gun, Jeollabuk-do; MHH	2*n* = 22	29.99 ± 0.49
DY1_6	Mt. Deokyu, Muju-gun, Jeollabuk-do; MHH	2*n* = 22	29.97 ± 0.05
DY2_1	Mt. Deokyu, Muju-gun, Jeollabuk-do; MHH	-	29.47 ± 0.09
DY2_2	Mt. Deokyu, Muju-gun, Jeollabuk-do; MHH	2*n* = 22	28.77 ± 0.65
DY2_3	Mt. Deokyu, Muju-gun, Jeollabuk-do; MHH	2*n* = 22	28.39 ± 0.33
DY2_4	Mt. Deokyu, Muju-gun, Jeollabuk-do; MHH	2*n* = 22	29.43 ± 0.39
DY2_5	Mt. Deokyu, Muju-gun, Jeollabuk-do; MHH	-	29.94 ± 0.85
DY3_1	Mt. Deokyu, Muju-gun, Jeollabuk-do; TSJ	2*n* = 22	29.31 ± 0.44
YD_1	Youngdong-gun, Chungcheongbuk-do; MHH	2*n* = 22	29.20 ± 0.98
YD_2	Youngdong-gun, Chungcheongbuk-do; MHH	2*n* = 22	28.38 ± 0.38
YD_3	Youngdong-gun, Chungcheongbuk-do; MHH	-	29.48 ± 0.49
YD_4	Youngdong-gun, Chungcheongbuk-do; MHH	-	28.26 ± 0.45
YD_5	Youngdong-gun, Chungcheongbuk-do; MHH	-	30.14 ± 0.11
HC_1	Hwacheon-gun, Gangwon-do; MHH	2*n* = 22	29.29 ± 0.68
HC_2	Hwacheon-gun, Gangwon-do; MHH	2*n* = 22	28.73 ± 0.31
HC_3	Hwacheon-gun, Gangwon-do; MHH	-	29.59 ± 0.33

Note: GPS coordinates, latitude, and longitude of the collected sites of threatened species, *Cypripedium japonicum*, are not indicated for protection purposes (permit no. 2021-17 and 2021-18 from the Hwacheon-gun, Mt. Deokyu, Muju-gun, and Youngdong-gun populations).

**Table 2:** New and previously published chromosome counting (2*n*) and genome size (1C/pg) data in the genus *Cypripedium*.

Taxon	2*n*	References	1C (pg)	References
*Cypripedium acaule* Aiton	20	[11]	44.84	[24]
*C. arietinum* R.Br.	20	[11]	-	-
*C. calceolus* L.	20	[11]	32.35	[24]
			41.05	[29]
*C. californicum* A.Gray	20	[30]	21.60	[30]
*C. candidum* Muhl. ex Willd.	20	[11]	-	-
*C. cordigerum* D.Don	20	[31]	-	-
*C. debile* Rchb.	20	[32]	-	-
*C. elegans* Rchb.	20	[11]	-	-
*C. fasciculatum* kellogg	20	[11]	-	-
*C. flavum* P.F.Hunt and Summerh.	20	[11]	36.41	[33]
*C. formosanum* Hayata	20, 30	[10]	28.50, 32.00	[24]
*C. guttatum* Sw.	20	[11]	-	-
*C. henryi* Rolfe	22	[32]	38.80	[24]
*C. himalaicum* Rolfe	20, 30	[11]	-	-
*C. japonicum* Thunb. *(population in Japan)*	20	[10]	-	-
*C. japonicum* Thunb. *(population in Korea and China)*	22	[12], Present study	28.38–30.14	Present study
*C. macranthos* Sw.	20, 22	[11]	37.40	[24]
*C. molle* Lindl.	20	[11]	4.14	[24]
*C. parviflorum* Salisb.	20	[11]	-	-
*C. parviflorum* var. *makasin* (Farw.) Sheviak	20	[11]	-	-
*C. parviflorum* var. *pubescens* (Willd.) O. W. Knight	20	[30]	32.40	[30]
*C. passerinum* Richardson	20	[11]	-	-
*C. plectrochilum* Franch.	20	[11]	-	-
*C. reginae* Walter	20	[11]	-	-
*C. segawae* Masam.	20	[10]	-	-
*C. shanxiense* S.C.Chen	20	[11]	-	-
*C. tibeticum* King ex Rolfe	20	[32]	-	-
*C. yatabeanum* Makino	20	[10]	-	-

**Table 3:** Karyotype analyses in the Korean endangered species, *Cypripedium japonicum*.

Collection Number (Population_Individual)	Absolute Chromosome Length (µm)											HCL^1^ ± S.D. (µm)	AsI ^2^ (%)	RI ^3^
	1	2	3	4	5	6	7	8	9	10	11			
DY1_1	14.57	13.49	12.47	9.94	9.81	9.31	8.67	7.71	6.27	5.25	4.58	102.07 ± 3.27	66.7	1.69
DY1_5	12.33	10.26	9.38	9.00	8.25	7.86	7.30	7.11	6.47	4.94	4.26	87.04 ± 2.30	59.9	1.84
DY2_2	12.52	10.69	9.28	9.43	8.27	6.78	8.56	7.31	6.22	5.38	4.72	89.16 ± 2.33	61.9	3.51

^1^ HCL: total haploid chromosome length; ^2^ AsI (Asymmetry Index): the proportion of all long arms to the total haploid karyotype length; ^3^ RI (Ratio Index): the ratio of the longest to the shortest chromosome.

## Data Availability

The data presented in this study will be available on request from the corresponding authors.
